# Diet and Physical Activity Apps: Perceived Effectiveness by App Users

**DOI:** 10.2196/mhealth.5114

**Published:** 2016-04-07

**Authors:** Qing Wang, Bjørg Egelandsdal, Gro V Amdam, Valerie L Almli, Marije Oostindjer

**Affiliations:** ^1^ Department of Chemistry, Biotechnology, and Food Science Norwegian University of Life Sciences Aas Norway; ^2^ Department of Ecology and Natural Resource Management Norwegian University of Life Sciences Aas Norway; ^3^ School of Life Sciences Arizona State University Tempe, AZ United States; ^4^ Nofima AS Aas Norway

**Keywords:** diet app, physical activity app, perceived effectiveness, behavioral changes

## Abstract

**Background:**

Diet and physical activity apps are two types of health apps that aim to promote healthy eating and energy expenditure through monitoring of dietary intake and physical activity. No clear evidence showing the effectiveness of using these apps to promote healthy eating and physical activity has been previously reported.

**Objective:**

This study aimed to identify how diet and physical activity (PA) apps affected their users. It also investigated if using apps was associated with changes in diet and PA.

**Methods:**

First, 3 semi-structured focus group discussions concerning app usability were conducted (15 app users and 8 nonusers; mean age 24.2 years, SD 6.4), including outcome measures such as motivations, experiences, opinions, and adherence. Results from the discussions were used to develop a questionnaire. The questionnaire, which contained questions about behavior changes, app usage, perceived effectiveness, and opinions of app usability, was answered by 500 Norwegians, with a mean age of 25.8 years (SD 5.1).

**Results:**

App users found diet and PA apps effective in promoting healthy eating and exercising. These apps affected their actions, health consciousness, and self-education about nutrition and PA; and were also a part of their social lives. Over half of the users perceived that apps were effective in assisting them to eat healthily and to exercise more. Diet apps were more effective when they were frequently used and over a long period of time, compared to infrequent or short-term use (*P*=.01 and *P*=.02, respectively). Users who used diet and PA apps, perceived apps as more effective than users who only used one type of app (all *P*<.05). App users were better at maintaining diet and PA behaviors than nonusers (all *P*<.05). Young adults found apps fun to use, but sometimes time consuming. They wanted apps to be designed to meet their personal expectations.

**Conclusions:**

App usage influenced action, consciousness, self-education about nutrition and PA, and social life. It facilitated maintaining a healthy diet and exercising more. Diet and PA apps of the future can be further strengthened by being tailored to meet personal needs.

## Introduction

Since the mainstream adoption of smartphones during the last decade, consumers have since had easy access to a tremendous amount of health information through websites, social media, and health apps [1]. Health apps provide information to users whenever and wherever they want, and are tools for users who have a goal to improve their health. Diet apps and physical activity (PA) apps are 2 types of health apps that aim to promote healthy eating and increased energy expenditure through monitoring dietary intake and PA. Using apps to affect eating behavior and PA behavior can be explained by the theory of planned behavior [2,3]. This theory shows that behavioral intention (eg, healthy eating, exercising) is driven by 3 constructs: attitudes towards the behavior, perceived behavioral control, and subjective norms. Attitudes are users’ positive or negative evaluations of self-performance of the behavior. Perceived behavioral control is users’ perceived ease or difficulty of performing the behavior. Subjective norms are users’ perceptions of the behavior. Using apps may influence users’ attitudes towards healthy eating or exercising, and it may relieve difficulties related to users engaging in healthy eating and exercise.

Many different types of diet and PA apps exist in app stores on different platforms. Diet/caloric intake apps and PA apps (fitness/training) are among the most popular in the "health and wellness” categories in app stores [4]. A diet app typically requires users to manually register what they eat each day. It converts food consumption into nutrition intake, summarizes results in plots and graphs, compares results with nutrition goals, offers nutrition and dieting information, and allows users to add their social network [5]. A PA app typically has GPS tracking to record physical activities, such as walking, jogging, and cycling. It also accurately records duration, frequency, and intensity of activities through an integrated gyroscope and/or accelerometer [6,7]. In addition, it calculates calorie expenditure, summarizes performance trends over time periods, and allows users to share their performance with friends on social networks.

Up to now, studies on diet and PA apps have evaluated the content of these apps and whether they were guided by relevant theory, or followed nutritional recommendations [8-10]. More research and evaluation is needed to show the perceived effectiveness of using these apps on healthy eating or increase in PA [11,12]. One approach is to evaluate how effective apps are from the users’ point of view, and if they believe that app usage in general, independent of their detailed construction, will actually result in an intended behavior. Perceived effectiveness has been used for app evaluation [5]. It presents the effectiveness of the information system (perceived by the users [13]). This perceived effectiveness, thereby, reflects the user’s self-assessment, and does not necessarily reflect actual effectiveness [14]. In general, previous studies evaluated health behavior change by using apps through qualitative methods [15] or only focused on one kind of app [16,17]. This study included both diet and PA apps, and evaluated perceived effectiveness through both qualitative and quantitative methods.

The objectives of this study were to identify how users perceive that they are affected by app use, and to investigate whether the use of apps was associated with improved diet and PA. Outcomes would indicate the potential of diet and PA apps for improving health.

## Methods

This study used a combination of qualitative and quantitative methods. Three semi-structured focus group discussions were conducted with 15 app users (2 groups) and 8 nonusers (1 group), with a mean age of 24.2 years (SD 6.4). Participants discussed motivations for, experiences with, opinions about, and adherence to using health apps. The discussion results were summarized for a number of key topics, which were transformed into a questionnaire. The resulting questionnaire (Multimedia Appendix 1) was answered by 500 Norwegians, with a mean age of 25.8 years (SD 5.1).

### Focus Group Discussions

Participants were students and staff at the Norwegian University of Life Sciences. They were recruited by email, and participated voluntarily. Selection of participants aimed to obtain a sufficient sample size of both app users and nonusers. Two focus group discussions with 2 male app users and 13 female app users, with an average age of 22.3 years (SD 7.3), were conducted and lasted 1.5 hours each. One focus group discussion with 6 male nonusers and 2 female nonusers, with a mean age of 24.8 years (SD 4.2), was conducted and lasted 1 hour. Female app users showed a higher interest in participating in focus group discussions, so there were more female app users than male app users in the focus groups. The 23 participants had 15 different university majors and lived in Akershus County and Oslo, near the university. Participants received monetary compensation for their participation (NOK 300/US $36). An experienced moderator led all 3 focus group discussions. In addition, an observer was present to take notes. The sessions were videotaped after consent was obtained from the participants.

Focus group discussions started with a general discussion about being healthy. Participants talked about methods they used to check health information and how they used health-related apps on a mobile phone, tablet, or computer. App operating systems were almost exclusively Android and iPhone OS. Users shared app usage motivation, goals, experiences, what they considered to be apps’ pros and cons, and expectations for future apps. Nonusers shared personal opinions about health apps, reasons and barriers for not using apps, and expectations for future apps. This completed the discussion of whether using health apps could help people keep healthy, and how to adapt future apps to meet the needs of users.

Focus group discussions were transcribed and translated from Norwegian to English. Key topics were defined through indexing and categorizing [18]. The key topics included duration of use, adherence to using apps, goals, motivations, perceived effectiveness, and barriers for using apps. Two types of health apps were mentioned most frequently: diet apps and PA apps. An app questionnaire was developed based on the key topics derived from the focus group discussions, focusing on diet and PA apps only.

### App Questionnaire

A cross-sectional Web-based questionnaire (Multimedia Appendix 1), aimed to assess dietary and PA changes and app usage among Norwegian young adults, was distributed in April 2015 through a market analysis company (Faktum Markedsanalyse AS, Oppegård, Norway). Participants were recruited by email from a national pool, and invited based on their age, which ranged from 18 to 35 years old; they had a balanced sex distribution; and half were health app users, while half were nonusers. Individuals participated voluntarily. Personal attributes of the participants are shown in Table 1.

The first question in the questionnaire was “Have you used diet apps or PA apps on a mobile phone, tablet, or computer during the last 12 months?” Participants who had app usage experience were categorized as users, and those who did not were categorized as nonusers. The questionnaire consisted of 4 parts: (1) questions about changes in dietary behavior and PA during the last 12 months; (2) questions about using diet apps and/or PA apps during the last 12 months; (3) questions about opinions about using apps; and (4) general personal attribute questions. Users answered all 4 parts. Nonusers answered parts 1, 3, and 4. This questionnaire took 10-20 minutes to complete, depending on whether subjects were users or nonusers and how many types of apps they used.

The first part of the questionnaire contained 10 questions about dietary behavior and PA changes. This section was presented first in the questionnaire before the app questions, to prevent participants from being prompted about the effectiveness of apps. Diet-related changes included paying attention to calorie information, choosing healthier food (low-fat products and mineral water instead of sweetened beverages), cooking at home more than buying ready-made meals, and searching for food or cooking information on the Internet or in books/magazines. Physical activity-related changes included becoming a gym member, having activity competitions, sharing information about PA on social networks, and searching for activity-related information on the Internet or in books/magazines. Participants indicated whether they showed these behaviors before April 2014 (ie, 1 year before the questionnaire) and whether they showed these behaviors in April 2015 (ie, when they answered the questionnaire). Four questions asked participants about their goals and efforts to improve their diet and increase their PA in the last 12 months. A five-point scale (a lot less, a little bit less, about the same, a little bit more, a lot more) was used to measure their changes in food consumption and PA. Two questions examined weight loss goals and weight change during the last 12 months.

The second part of the questionnaire contained 12 questions. It first introduced general concepts of the apps and gave an example of a diet app (“myfittnesspal”) and an example of a PA app (“Moves”) [19,20]. Both apps were available for Android and iOS. Then, participants were asked about their duration and frequency of using the app in both the first and last month (if they stopped using the app before the questionnaire), goals (single choice) and motivations for using the apps, and perceived effectiveness of using diet and PA apps. The diet apps’ effectiveness in assisting users to eat more low-fat alternatives in place of dairy products, eat more fruit and vegetables, eat less sausages, drink less sweetened beverages, eat less fast food, and choose healthier food products was evaluated. These diet changes were included based on the Nordic Nutrition Recommendations 2012, 5th edition [21]. The PA apps’ effectiveness in assisting users to increase time spent on exercising, exercise more often, increase exercise intensity, and diversify their activities was measured using a 4-point scale (very effective, somewhat effective, slightly effective, or not effective).

The third part of the questionnaire contained 15 questions. A 7-point agree/disagree scale (disagree strongly, disagree moderately, disagree slightly, neutral, agree slightly, agree moderately, or agree strongly) was used to measure participants’ opinions about apps and barriers for using those apps. Barriers included “it is hard to obtain information from apps,” “it is time consuming to use apps,” and “the apps do not fit personal expectations.”

The fourth part of the questionnaire contained questions about gender, age, living region, weight, height, marital status, education, employment situation, yearly income, and food and health concerns. Food and health concerns were examined with the questions, “I am concerned about getting a lot of… (calories/fat/sugar) in my food” and “I am concerned about gaining weight” using a 5-point scale, from “I am extremely concerned” (5) to “I am not concerned at all” (1) [22]. Based on these questions, the survey had good reliability of responses (Cronbach alpha =.85).

The questionnaire was pretested by 6 food researchers and three master’s students from the Department of Chemistry, Biotechnology, and Food Science, Norwegian University of Life Sciences. Small amendments were made to ensure that the questionnaire was clear, concise, and user-friendly.

### Analysis of Questionnaire Data

App usage among app users was described by 4 factors from the questionnaire data: user type (users who used both diet app and PA apps; users who used only one type of apps); duration (0-1 months, 1-6 months, 6-12 months, or over 12 months); adherence (less frequently, same frequency, or more frequently); and goals. The goals for using diet apps were categorized into 4 types: to track food intake, to facilitate weight loss, to be healthy, and other goals. The goals for using PA apps were categorized into 4 types: to track PA, to do more PA, to facilitate weight loss, and other goals. The perceived effectiveness of using apps was categorized into effective, not effective, and do not know. The behavior changes were summarized into 4 categories (maintain, develop, give up, or never have the behavior) based on whether people had the behavior before April 2014 and whether they still had the behavior in April 2015. Food and health concern scores were calculated and participants were divided into 2 groups (high or low food, and health concern). Weight status (underweight, normal, overweight, or obese) was categorized based on body mass index (BMI) calculations from the questionnaire data.

All statistical analyses were performed using the statistical program R and R Commander version 3.2. Data were checked for model assumptions. Multinomial logistic models (MLM) identified associations between perceived effectiveness of using apps and app usage. The model was perceived effectiveness = user type (use both apps, or use only one type of apps) + duration + adherence + goals. MLM also identified associations between dietary behavior changes and app usage, association between PA changes and app usage, and association between weight change and app usage. The model was behavior changes = user type (use both apps, use only diet apps, use only PA apps, or nonusers) + food and health concerns + weight status. Chi-square tests identified differences among app user groups. Associations between app usage and food consumption changes were identified by chi-square tests to explain weight changes among app users. Chi-square tests also identified associations between app usage and opinions about apps.

**Table 1:** Personal attributes of questionnaire participants (N=500).

Variable		%
**Sex**	Male	50.0
	Female	50.0
**BMI** ^a^	Underweight (<18.5)	4.4
	Normal weight (18.5-24.9)	57.9
	Overweight (25-29.9)	24.0
	Obese (>30)	13.8
**Living region**	Northern Norway	8.7
	Mid Norway	13.3
	Western Norway	28.0
	Southern Norway	8.5
	Eastern Norway	41.7
**Employment situation**	Employed for wages	45.4
	Self-employed	3.8
	Unemployed	5.2
	Staying at home	3.4
	Student	35.6
	In the military	1.2
	Unable to work	5.4
**Food and health concerns**	High concern about food and health	16.8
	Low concern about food and health	83.2
**Highest education**	Primary school	13.2
	Secondary school	47.0
	College or university up to bachelor	28.2
	College or university up to master or PhD	11.6
**Yearly income**	0-200,000 NOK^b^	44.6
	200,000-400,000 NOK	26.2
	400,000-600,000 NOK	18.0
	600,000-800,000 NOK	6.0
	800,000-1,000,000 NOK	1.8
	>1,000,000 NOK	3.4
**Marital status**	Not married, without children	59.2
	Not married, with children	6.6
	Married or domestic partnership, without children	16.2
	Married or domestic partnership, with children	15.0
	Separated/Divorced/Widowed, without children	1.4
	Separated/Divorced/Widowed, with children	1.6

^a^ BMI: body mass index

^b^ NOK: Norwegian Kroner

## Results

### Focus Group Outcomes: A Model of Apps’ Effects on Users

**Figure 1:**
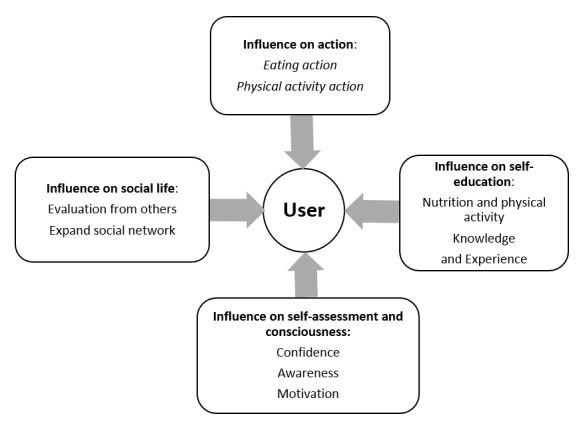
Qualitative influences perceived by app users based on focus groups. Four themes were summarized from focus group discussions.

A model was summarized from the focus group discussions (Figure 1). It showed the influences of apps on users, according to the focus groups, categorized into 4 themes. Overall, apps offered an overview of how much one ate and exercised. For instance, diet app users obtained nutritional information about their daily consumptions of calories, carbohydrates, fat, and protein. These apps summarized and evaluated users’ food intake. For example, one user said the following:

The app told me if I ate too few carbs relative to fat or protein intake.

Female, 21 years

Thus, by knowing their nutritional intake, users could adjust their eating to reach their goal of a balanced diet. Meanwhile, through this process, users gained experience and knowledge of nutrition and healthy eating. Using apps influenced self-assessment of diet, PA, and consciousness. Some users reported that they felt good about themselves because of their app usage, while other users felt stressed about using diet apps, mainly because it was time-consuming to register all the food items they consumed. Users felt that using apps could lead to higher awareness of the nutritional content of food, and higher awareness of and motivation for healthy eating and exercising. There were 2 examples given by users, who summarized the functions of diet and PA apps:

[You get] inspiration, information, [and] motivation to make healthier choices and confirmation that you have made the right choices, and guidance and tips about new food.

Female, 19 years

I have used an exercise app to get an overview of my activity. I used it to get some graphs and so on. It was motivating.

Male, 24 years

Influence on social life was another key point in the discussion. Users received positive or negative feedback from their friends or family. They could easily share the outcomes from apps on the Internet, especially those from the PA apps.

Results of training are fun to share.

Male, 24 years

Users could also be enrolled in a social network using apps, such as weight loss or dieting groups. They could either make new friends or strengthen relationships with old friends or family. Sharing diet or exercise outcomes on the Internet became one important motivation for participants to continue using apps.

### Questionnaire Outcomes: Perceived Effectiveness of Using Diet Apps

Overall, 186 diet app users and 192 PA app users answered the questionnaire, among whom 128 used both diet and PA apps. In general, diet and PA app users felt that apps were effective to facilitate their healthy food intake and activities. More than half of the diet app users felt that diet apps effectively assisted them to eat more fruit and vegetables (133/186, 71.5%), eat less fast food (117/186, 62.9%), choose healthier food products (117/186, 62.9%), and drink less sweetened beverages (106/186, 57.0%). Nearly half of diet app users found diet apps effective in assisting them to eat more low-fat dairy products (91/186, 48.9%) and less sausages (88/186, 47.3%). The majority of PA app users felt that PA apps effectively assisted them to exercise more often (144/192, 75.0%) and increase the intensity of exercises (139/192, 72.4%). More than half of the PA app users found that PA apps were effective in assisting them to increase time spent exercising (129/192, 67.2%) and diversify activities (106/192, 55.2%).

### Perceived Effectiveness of Diet Apps Influenced by User Type, Duration, and Adherence

User type, duration, and adherence influenced perceived effectiveness of eating less sausages (*P*=.03), eating more fruit and vegetables (*P*=.01), and eating more low-fat dairy (*P*=.02), respectively. Goals did not influence perceived effectiveness. App usage, duration, adherence, and goals did not influence users’ perceived effectiveness of diet apps for choosing healthier food products, drinking less sweetened beverages, or eating less fast food.

**Figure 2:**
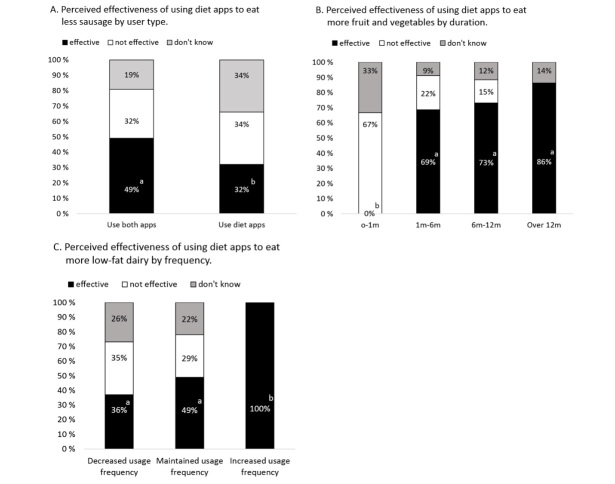
Percentages of different diet app user categories and their evaluation of the effectiveness of using diet apps to assist their food intake.

Users of both diet and PA apps had a higher probability of reporting that diet apps effectively assisted them to eat less sausages than users who only used diet apps, χ^2^_1_=4.2, *P*=.04 (Figure 2, Part A). Duration was associated with perceived effectiveness of eating more fruit and vegetables. Users who used diet apps for more than one month had a higher probability of reporting that apps were effective in assisting them to eat more fruit and vegetables than users who used diet apps for less than one month (all *P*<.05, Figure 2, Part B). Adherence was associated with perceived effectiveness of eating more low-fat dairy. Diet app users, who had increased the frequency of using apps in the past 12 months, had a higher probability of reporting that apps were effective in assisting them to eat more low-fat dairy than users who decreased their app usage frequency, χ^2^_1_=11.1, *P*<.001, or users who maintained the same frequency of using apps, χ^2^_1_=7.4, *P*=.007 (Figure 2, Part C).

### Perceived Effectiveness of Using PA Apps Influenced by App Usage and Goals

App usage influenced the perceived effectiveness of diversifying physical activities (*P*=.003). Duration of and adherence to using apps did not influence users’ perceived effectiveness of PA apps. Goals influenced perceived effectiveness for increasing time spent exercising, exercising more often, increasing intensity of exercises, and diversifying activities (all *P*<.05).

**Figure 3:**
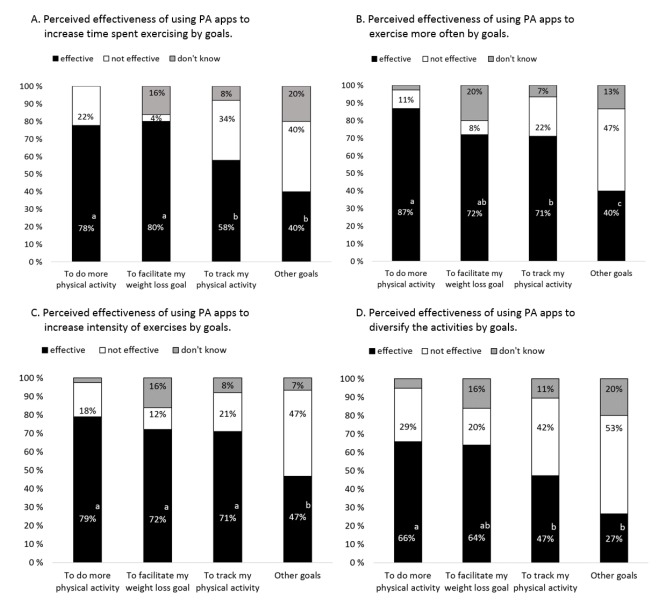
Percentages of PA app users with different goals and their evaluation of the effectiveness of using PA apps to assist their physical activities.

App usage was associated with perceived effectiveness of diversifying activities. More users of both diet and PA apps reported that PA apps effectively assisted them to diversify activities than did those who used only PA apps, χ^2^_1_=12.2, *P*<.001. Goals were associated with perceived effectiveness of using PA apps (Figure 3). PA app users with a goal to do more PA or to lose weight had a higher probability of reporting that apps were effective in assisting them to increase time spent on exercising, than did users who only wanted to track their PA (*P*=.009 and *P*=.046, Figure 3, Part A). PA app users who had a goal to do more PA had a higher probability of reporting that apps were effective in assisting them to exercise more often or to diversify their activities than users who had a goal to track their PA (both *P*=.02) or who had other goals (*P*<.001 and *P*=.005, Figure 3, Parts B and D). More PA app users who had a goal to do more PA, to reach a weight loss goal, or to track PA, reported that apps were effective in assisting them to increase the intensity of exercises than did users who had other goals (all *P*<.05, Figure 3, Part C).

### Dietary and Physical Activity Behavior Changes and Weight Change Associated With App Usage

#### Dietary Behavior Changes Influenced by App Usage

**Figure 4:**
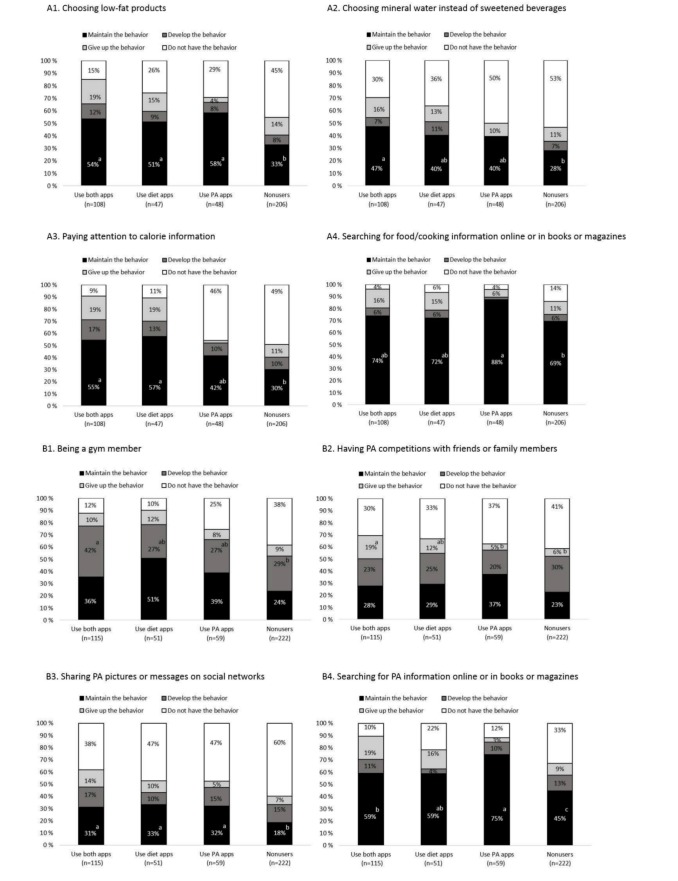
Percentages of dietary behavior change and physical activity behavior change among different participants (use both apps, use diet apps, use PA apps, and nonusers). A1-A4. Dietary behavior changes. B1-B4. Physical activity behavior changes.

App usage was associated with the following dietary behavior changes: choosing low-fat products, choosing mineral water instead of sweetened beverages, paying attention to calorie information, and searching for information about food and cooking (all *P*<.05, Figure 4A). App usage did not influence the behavior change of cooking at home instead of buying ready-made meals. Food and health concerns were associated with paying attention to calorie information and cooking at home instead of buying ready-made meals (*P*<.001 and *P*=.03). Weight status was not associated with dietary behavior changes (all *P*>.15).

App users had a higher probability of maintaining the behavior of choosing low-fat products instead of ordinary products compared to nonusers (all *P*<.05, Figure 4A1). Users of both diet and PA apps had a higher probability of maintaining the behavior of choosing mineral water instead of sweetened beverages compared to nonusers (χ^2^_1_=11.4, *P*<.001, Figure 4A2). Diet app users—who used both diet and PA apps or only diet apps—had a higher probability of maintaining the behavior of paying attention to calorie information than nonusers (both *P*<.001, Figure 4A3). Those who used only PA apps had a higher probability of maintaining the behavior of searching for information about food or cooking on the Internet, or in books or magazines, than nonusers (χ^2^_1_=6.4, *P*=.01, Figure 4A4).

#### Physical Activity Behavior Changes Influenced by App Usage

App usage was associated with changes in PA including becoming a gym member, having competitions with friends and family members, sharing pictures or messages related to exercises on a social network, and searching for PA information on the Internet or in books or magazines (all *P*<.05, Figure 4B). Food and health concerns, as well as weight status, were associated with the PA behavior change of having competitions with friends or family members (*P*=.009 and *P*=.02). Food and health concerns, and weight status, were not associated with the other PA behavior changes.

Users of both diet and PA apps had a higher probability of becoming gym members than nonusers (χ^2^_1_=7.2, *P*=.007, Figure 4B1), and a higher probability of giving up having PA (eg, running, skiing) competitions with friends or family members than nonusers (χ^2^_1_=6.3, *P*=0.01) and those who only used PA apps (χ^2^_1_=13.1, *P*<.001, Figure 4B2). They also had a higher probability of continuing to share pictures or messages related to their exercises on a social network than nonusers (χ^2^_1_=7.1, *P*=.007, Figure 4B3). Those who used only PA apps had a higher probability of continuing to search for PA information on the Internet, or in books or magazines, than those who used both diet and PA apps (χ^2^_1_=4.1, *P*=.04) or nonusers (χ^2^_1_=16.8, *P*<.001, Figure 4B4).

#### Weight Change Influenced by App Usage

App users and nonusers differed in their weight change (*P*=.001). Food and health concerns and weight status did not affect weight change. Those who used both diet and PA apps and those who used only diet apps had a higher probability of weight loss during the last 12 months compared to nonusers (*P*<.001 and *P*=.01) and users who used only PA apps (*P*=.001 and *P*=.03). Diet app users ate more fruit and vegetables and a lower total amount of food during the last 12 months compared to PA app users and nonusers (*P*=.04 and *P*=.002, respectively). There was no significant difference in low-fat food, processed meat, sweetened beverage, and fast food consumption between PA app users and nonusers (all *P*>.1).

### Opinions About Apps

Both app users and nonusers provided their opinions about the apps (Table 2). In sum, 339 out of 500 participants (67.8%) thought that mobile phones, tablets, or computers were easy for them to use and they liked using them; and 319 out of 500 participants (63.8%) felt apps were not hard for them to understand. Half of the participants thought it was not hard to obtain information from apps, and only 143 out of 500 participants (28.6%) thought it was time consuming to use these apps. In total, 160 out of 500 participants (32.0%) felt it was fun to use apps.

Generally, the app users had positive opinions about using health apps. Their opinions were more positive than nonusers’ perceptions of app usage. Comparing to nonusers, there were more app users who agreed with opinions that they were concerned about health, and so they wanted to use health apps and found it fun to use them (both *P*<.001). More app users disagreed with opinions that apps could not help them to be healthy, that it was hard to get information from apps, that it was time consuming to use apps, or that they could not find an app that fit their expectations, compared to nonusers (all *P*<.001).

**Table 2:** Opinions about apps—percentages of disagreement/agreement with nine statements about health apps (N=500).

Opinions	Disagree strongly	Disagree moderately	Disagree slightly	Neutral	Agree slightly	Agree moderately	Agree strongly
I like to use smartphones, tablets, or computers.	6.4%	3.8%	4.2%	17.8%	12.2%	15.8%	39.8%
It is easy for me to use smartphones, tablets, or computers.	3.6%	3.8%	3.4%	14.4%	9.4%	13.2%	52.2%
It is hard for me to understand how health-related apps work on smartphones, tablets, or computers.	37.0%	16.6%	10.2%	25.6%	6.2%	2.6%	1.8%
I am concerned about my health, so I want to use health-related apps.	28.8%	13.8%	7.4%	30.2%	12.0%	5.8%	2.0%
I think health-related apps cannot help me to be healthy.	14.8%	13.4%	18.0%	31.0%	9.2%	8.0%	5.6%
It is hard for me to get information from health-related apps.	17.2%	19.2%	13.6%	38.2%	7.6%	1.6%	2.6%
It is time consuming for me to use health-related apps.	11.2%	11.6%	15.2%	33.4%	18.0%	6.6%	4.0%
I find it fun to use health-related apps.	10.6%	7.6%	9.0%	40.8%	15.8%	11.6%	4.6%
I cannot find a health-related app that fits my expectations.	12.2%	9.2%	10.8%	48.4%	11.2%	4.8%	3.4%

## Discussion

### Principle Findings

This study suggests that users find diet and PA apps effective in promoting healthy eating and more exercise through effects on their actions, health consciousness, self-education about nutrition and PA, and social life. Apps were particularly effective when they were used frequently and over a long period (eg, more than 1 month). App usage was also associated with actual self-reported behavior, particularly maintenance of healthy behaviors, and also, depending on the goal, adoption of new behaviors in the case of PA apps.

In the focus group, users and nonusers discussed and evaluated the apps’ influence on diet and PA. Users reported that using apps influenced eating and exercising. Based on responses to the questionnaire, they perceived that they ate healthier foods and exercised more when using apps. A previous qualitative study showed that users considered an app’s ability to record and track behavior and goals as valuable [23]. By recording and tracking food intake and physical activities, apps give feedback to users on how well they are doing in reaching their goals. One study reported that feedback significantly increased users’ motivation to engage in PA [24]. Apps act as a reminder or evaluator for users. They also give suggestions and alternatives related to dieting and exercising that aim to help users achieve their goals. In the focus group discussions, users felt more confident about themselves when they experienced success in healthy eating and increased exercising. Frequent use over time can result in positive evaluation of self-performance, and in response, an improved attitude towards the behavior (in this case healthier eating or increased exercising), particularly when the app has options to show users their progress over time. Increased knowledge and awareness, which often were brought up in the focus groups, can make it easier for users to perform a behavior, and thus increase perceived behavioral control. Users also experienced interactions between social networks and app usage, which may in turn affect social norms. They received both positive and negative comments and feedback from friends and family members, and sometimes even used apps together with friends, which facilitated sharing of outcomes. Based on the theory of planned behavior, using apps influenced all three constructs (attitudes towards the behavior, perceived behavioral control, and subjective norms), which strengthened the behavior intention. The stronger the intention, the more likely it was that users would execute a healthy behavior [25]. In this study, app users perceived that apps were effective in facilitating their food intake and activity. Results from this study showed that diet apps could be effective in promoting users to follow the Nordic Nutrition Recommendations, and PA apps could be effective in promoting users to increase duration, frequency, intensity, and diversity of exercise. Using apps strengthened users’ intentions and behavior performance.

The findings of this study support the concept that app usage can expand eHealth literacy. eHealth literacy reflects people’s ability to seek, find, understand, and appraise health information from electronic sources and apply that knowledge to make a health-related decision [26]. Users explored eHealth information through the apps. By seeking, understanding, and appraising eHealth information, they processed it to guide their actions. At the same time, limited eHealth literacy can preclude some populations from accessing health information. Prior research has shown that individuals’ education, health status, and motivation influences eHealth literacy [27]. Furthermore, younger and more educated people have higher eHealth literacy than their counterparts [28]. However, in general, users find apps easy and convenient to use [29,30]. These findings coincide with those of this study, in that half of the survey population thought it was not hard to obtain information from apps.

Since using apps could strengthen behavioral intention and expand eHealth literacy, this study also examined whether using apps led to self-reported health behavior changes during the last 12 months. Health behavior change is a central objective of health promotion, and new health behaviors are often not maintained [31,32]. Results of the questionnaire showed that app usage was associated with maintenance of healthy behaviors. App users continued to choose low-fat products instead of high-fat alternatives and mineral water instead of sweetened beverages, and continued to look for diet-related and PA-related information more often than the nonusers. Using apps advanced self-regulation skills and ability, and supported users to engage in healthy behaviors [33]. In addition, over 66.7% (128 out of 192) of the app users used both diet and PA apps, and monitored both food intake and energy output. These individuals maintained health behaviors better than those who only used diet or PA apps. Future studies could provide details on the role of combined app usage in changing health behaviors, to give more specific advice.

In this study, there was no direct evidence showing a relationship between app users’ perceived effectiveness of using apps and their actual health behavior changes. This study had a few examples showing a weak link between perceptions and behavior. For example, more than half of the app users perceived that PA apps effectively assisted them to be active; meanwhile, this group of people had a higher probability of becoming a gym member as an actual behavior. However, perceptions may not always match behaviors. The relationship between perceived effectiveness of using apps and actual behavior change needs further evaluation.

Users reacted in various ways toward the apps. According to the questionnaire, 97 out of 500 participants (19.4%) agreed that they could not find an app that met their expectations. Further development of diet and PA apps could involve tailoring to match requirements on a personal or subgroup level, such as for teenagers, young adults, middle-aged adults, or older adults. These subgroups differ in knowledge, experience, health situations, and goals. Each user has individual needs, so personalization of apps is necessary. Tailoring apps to meet personal needs has been discussed and suggested in previous studies [23,34,35]. In this study, in the focus group discussions, some users complained that they had difficulty finding Norwegian brands and foods in diet apps, since most were not developed based on the Norwegian food market. Both users and nonusers mentioned that tailoring apps to fit personal interests would be a good idea for the future development of apps. Thus, users would benefit more if apps were tailored to their expectations and personal needs. Meanwhile, since nonusers’ perceptions of app usage were less positive than users in this study, tailoring apps to fit nonusers’ needs may increase their interest in using apps.

This study evaluated perceived effectiveness and self-reported behavior changes associated with app usage through a questionnaire. It revealed the effects of apps on healthy eating and exercising; however, these effects were not validated in a randomized controlled trial. Future studies should evaluate the strengths of the reported effects in randomized controlled trials with adequately powered sample sizes. The sample population in this study may be a limitation, and larger sample sizes should be implemented in future work.

### Conclusions

Using diet and PA apps influenced actions, consciousness, self-education about nutrition and PA, and social lives of users. App usage facilitated healthy eating and increased exercising, as well as the maintenance of healthy behaviors. The apps were considered fun to use; however, some (eg, dietary apps) were time-consuming. Future apps could be tailored to meet personal needs, and future studies could use app tracking data to measure actual food consumption and PA changes rather than perceived changes through self-reports.

## Appendix

Multimedia Appendix 1 Study questionnaire.

## Abbreviations

PA: physical activity
