# Supplementary material for: Diet and Physical Activity Apps: Perceived Effectiveness by App Users
Source: JMIR Mhealth Uhealth. 2016 Apr 7;4(2):e33. doi: 10.2196/mhealth.5114 (PMC4840256; doi:10.2196/mhealth.5114)
Supplement: Multimedia Appendix 1 [file mhealth_v4i2e33_app1.pdf]

App Questionnaire

1. Have you used diet apps, or physical activity apps on a mobile phone, tablet, or computer during the last 12 months?

a. Yes, I have used at least one of the three apps during the last 12 months, and I am currently using it/them.

b. Yes, I have used at least one of the three apps during the last 12 months, but I am not currently using it/them.

c. No, I have used none of the three apps during the last 12 months, but I know of at least one of them.

d. No, I have used none of the three apps during the last 12 months, and I know of none of them.

Behavior questions

Answer the following questions based on your normal routine during the last 12 months.

2. Here are statements about food behavior. Please choose the answers that fit you the best.

	I did this prior to the last 12 months, but not in the last 12 months	I did this prior to the last 12 months, as well as in the last 12 months.	I did not do this prior to the last 12 months, but I started to do it in the last 12 months.	I did not do this prior to the last 12 months, nor in the last 12 months.	I do not know
I paid attention to calorie information.					
I chose low-fat products instead of ordinary products.					
I chose mineral water instead of sweetened beverages.					
I cooked at home instead of buying ready-made meals.					
I searched for information about food or cooking on the Internet or in books or magazines.					

3. Here are statements about getting fit. Please choose the answers that fit you the best.

	I did this prior to the last 12 months, but not in the last 12 months	I did this prior to the last 12 months, as well as in the last 12 months.	I did not do this prior to the last 12 months, but I started to do it in the last 12 months.	I did not do this prior to the last 12 months, nor in the last 12 months.	I do not know
I was a member of a gym.					
I had physical activity (eg, running, skiing) competitions with friends or family members.					
I shared pictures or messages related to my exercises on my social networks (eg, Facebook, Twitter).					
I searched for information about physical activity on the Internet or in books or magazines.					
I searched for information about weight loss on the Internet or in books or magazines.					

4. Did your weight change during the last 12 months?

I lost weight. (If this answer was chosen, an additional question was asked about how many kg they had lost.)______kg.

I gained weight. (If this answer was chosen, an additional question was asked about how many kg they had gained.)______kg.

My weight did not change.

5. Did you have a specific goal to lose weight during the last 12 months?

   Yes

   No

6. Did you have a specific goal to improve your diet during the last 12 months?

   Yes

   No (skip the next question)

If you had a goal to improve your diet,

7. what methods did you try to improve your diet during the last 12 months? (Single choice or multiple choices)

   Followed specific diets

   Consulted dietitians

   Attended weight-loss courses

   Used diet apps

   Learned cooking or healthy food choices from TV, books, or the Internet.

   Other ______ (Fill in)

8. How did your food intake change during the last 12 months?

A lot less

A little bit less

About the same

A little bit more

A lot more

I do not eat this kind of food

Total amount of food per day

Fruit and vegetables

Low-fat products

Processed meat (anything cured, smoked, or bought from a deli)

Beverages with added sugar

Fast food (eg, McDonald's)

9. Did you have a specific goal to increase physical activity during the last 12 months?

   Yes

   No (skip the next question)

If you had a goal to increase physical activity,

10. what methods did you try to increase physical activity during the last 12 months? (Single choice or multiple choices)

    Went to a gym

    Consulted personal trainers

    Used physical activity apps

Joined sports clubs

Participated in sport competitions such as a marathon

Used a private exercise room

Other ______ (Fill in)

11. How did your physical activity change during the last 12 months?

A lot less

A little bit less

About the same

A little bit more

A lot more

I do not do this kind of physical activity

Physical activity in general

Walking

Active involvement in games and sports with children or walking domestic animals

Running

Fast cycling, skiing, or swimming

Football, volleyball, hockey, or other competitive sports

Exercise in a gym

Other activity______ (Fill in)

App questions

Section 1: diet apps

Here is <u>an example</u> of a diet app that enables users to track dietary intake, and gives relevant information about food and diet.

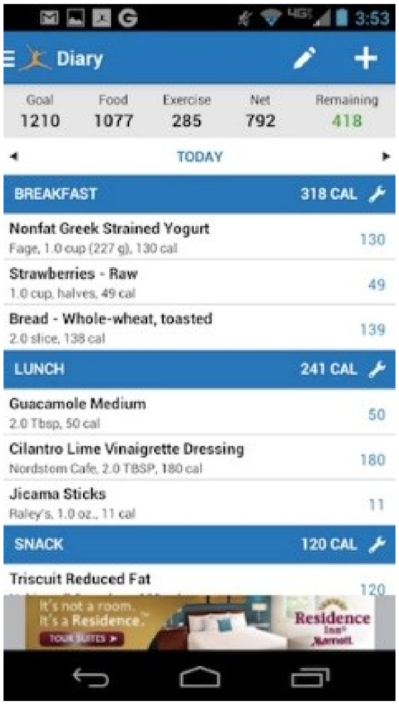

12. Have you used a diet app (on a mobile phone, tablet, or computer) during the last 12 months?

a. Yes, I have used a diet app during the last 12 months, and I am currently using it.

b. Yes, I used a diet app during the last 12 months, but I am not currently using it.

c. No, I know about diet apps, but I do not use them. (If participants choose this answer, they should jump to section 2.)

d. No, I do not know what a diet app is. (If participants choose this answer, they should jump to section 2.)

13. How long have you used a diet app? ______months

Please specify the period that you have used the app: (Fill in the sentence that fits you best)

From ____ (month), _____ (year) to now

Or

From ____ (month), _____ (year) to ____ (month), _____ (year)

Please think about the period that you use/used the diet app, and answer the following two questions.

14. How often did you use the diet app **during the first month after you started using it**?

    More than once per day

    1 time per day

    1-6 times per week

    1-3 times per month

    Less than 1 time per month

15. How often did you use the diet app **during the last month**?

    More than once per day

    1 time per day

    1-6 times per week

    1-3 times per month

    Less than 1 time per month

16. To what extent do you agree with the following statements? Please choose the answers that fit you the best.

Disagree strongly

Disagree moderately

Disagree slightly

Neutral

Agree slightly

Agree moderately

Agree strongly

It is easy for me to understand how the diet app works.

It is time consuming for me to use the diet app.

It is easy for me to get information from the diet app.

It is boring to use the diet app.

It is easy for me to reach my goal by using the diet app.

17. What is your main goal when using diet apps?

    To track my calorie intake

    To check nutrients in my food

    To check information because I have allergies, celiac disease, or other food-related health issues.

    To get an overview of my food consumption

To facilitate my grocery shopping

To facilitate my weight loss goal

To facilitate my work (dietitian, sports coach, etc)

To help me to eat healthier in general

Other _______ (Fill in)

18. What are your motivations for using a diet app to achieve your goal? Please choose the answers that fit you the best.

Disagree strongly

Disagree moderately

Disagree slightly

Neutral

Agree slightly

Agree moderately

Agree strongly

I think it is easy to obtain food information from the diet app.

I need to pay attention to my diet, because my family has disease history, (eg, cardiovascular disease or cancer).

I want to have diet competitions with friends or family members.

I want to share my diet on social networks.

Other _______ (Fill in)

19. How effective has the diet app been in assisting your diet?

Very effective

Somewhat effective

Slightly effective

Not effective

I do not know

To eat more low-fat dairy alternatives

To eat more fruit and vegetables

To eat less sausages

To drink less sweetened beverages

To eat less fast food, which is typically high in salt and saturated fat

To choose more healthy food products, such as food products with a Keyhole label

Comments _______ (Fill in)

20. How effective is using the app to reach your goal, compared to these methods?

Very effective

Somewhat effective

Slightly effective

Not effective

I do not know

Using the app is …. compared to specific diets.

Using the app is …. compared to weight-loss courses.

Using the app is …. compared to learning cooking or healthy food choices from TV, books, or the Internet.

Section 2: physical activity app

Here is <u>an example</u> of a physical activity app that enables users to track physical activity throughout the day, or track physical activity during specific events such as running, biking, or aerobics, and gives relevant information about exercise.

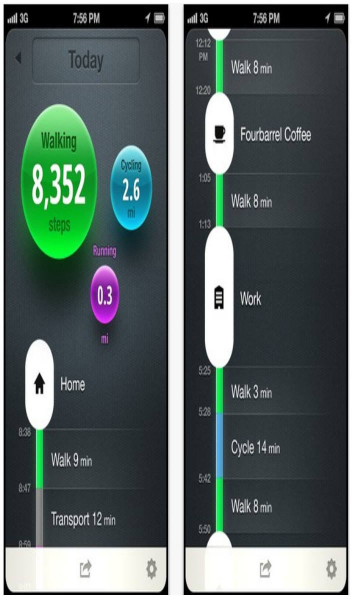

21. Have you used a physical activity app (on a mobile phone, tablet, or computer) during the last 12 months?

a. Yes, I have used a physical activity app during the last 12 months, and I am currently using it.

b. Yes, I used a physical activity app during the last 12 months, but I am not currently using it.

c. No, I know about physical activity apps, but I do not use them. (If participants choose this answer, they should jump to section 3.)

d. No, I do not know what a physical activity app is. (If participants choose this answer, they should jump to section 3.)

22. How long have you used a physical activity app? ______months

Please specify the period that you have used the app: (Fill in the sentence that fits you best)

From ____ (month), _____ (year) to now

Or

From ____ (month), _____ (year) to ____ (month), _____ (year)

Please think about the period that you use/used the physical activity app, and answer the following two questions.

23. How often did you use the physical activity app **during the first month after you started using it**?

    More than once per day

    1 time per day

    1-6 times per week

    1-3 times per month

    Less than 1 time per month

24. How often did you use the physical activity app **during the last month**?

    More than once per day

    1 time per day

    1-6 times per week

    1-3 times per month

    Less than 1 time per month

25. To what extent do you agree with the following statements? Please choose the answers that fit you the best.

Disagree strongly

Disagree moderately

Disagree slightly

Neutral

Agree slightly

Agree moderately

Agree strongly

It is easy for me to understand how the physical activity app works.

It is time consuming for me to use the physical activity app.

It is easy for me to get information from the physical activity app.

It is boring to use the physical activity app.

It is easy for me to reach my goal by using the physical activity app.

26. What is your main goal when using the physical activity app?

    To track my physical activity

    To do more physical activity

    To facilitate my weight loss goal

    To facilitate my work (dietitian, sports coach, etc)

    Other _______ (Fill in)

27. What are your motivations to use physical activity apps to achieve your goal? Please choose the answers that fit you the best.

Disagree strongly

Disagree moderately

Disagree slightly

Neutral

Agree slightly

Agree moderately

Agree strongly

I have health issues that can be managed through physical activity.

I have a high risk of disease due to family history (eg, cardiovascular disease or cancer).

I want my body to be in good shape.

I want to have physical activity competitions with friends or family members.

I want to share my physical activity on social networks.

Other _______ (Fill in)

28. How effective has the physical activity app been in assisting your physical activity?

Very effective

Somewhat effective

Slightly effective

Not effective

I do not know

To increase time spent exercising

To exercise more often

To increase intensity of exercises

To diversity the activities

Any comments _______ (Fill in)

29. How effective is using the app to reach your goal, compared to these methods?

Very effective

Somewhat effective

Slightly effective

Not effective

I do not know

Using the app is …… compared to consulting personal trainers.

Using app the is …… compared to joining sports clubs.

Using the app is …… compared to using private exercise rooms.

30. How did using the physical activity app change your frequency of exercising in number of days? Choose the sentence that fits you the best and fill in the number of days.

After using the physical activity app, I exercised__________ less days per week than before.
After using the physical activity app, I exercised__________ more days per week than before.
The number of days that I exercised did not change.

31. How did using the physical activity app change the number of hours that you exercised? Choose one sentence that fits you the best and fill in the number of hours.

After using the physical activity app, I exercised__________ hours less per session.
After using the physical activity app, I exercised__________ hours more per session.
The number of hours that I exercised did not change.

App usability questions

32. What are your opinions about new technology and health-related apps (such as diet apps, and physical activity apps)? Please choose the answers that fit you the best.

Disagree strongly

Disagree moderately

Disagree slightly

Neutral

Agree slightly

Agree moderately

Agree strongly

I like to use mobile phones, tablets, or computers.

It is easy for me to use mobile phones, tablets, or computers.

It is hard for me to understand how health-related apps work on mobile phones, tablets, or computers.

I am concerned about my health, so I want to use health-related apps.

I think health-related apps cannot help me to be healthy.

It is hard for me to get information from health-related apps.

It is time consuming for me to use health-related apps.

I find it fun to use health-related apps.

I cannot find a health-related app that fits my expectations.

Comments _______ (Fill in)

33. What is your main problem with health-related apps? (Open question)

General questions

34. How do you identify your gender?
    Male
    Female

35. What is your age? (Dropdown menu with possible ages, 18-35)

36. What are the first 3 digits of your postcode? ___________

37. My height is (fill in): _________ centimeters

38. My weight is (fill in): _________ kilograms

39. What is your marital status?
    Not married, without child/children.
    Not married, with child/children.
    Married or domestic partnership, without child/children.
    Married or domestic partnership, with child/children.

Separated/divorced, without child/children.
Separated/divorced, with child/children.
Widower/widowed, without child/children.
Widower/widowed, with child/children.

40. Are you currently…?
Employed for wages
Self-employed
Jobless
Staying at home
A student
In the military
Unable to work

41. What is your highest level of education attained?
Primary (Barneskolen/Ungdomsskolen)
Secondary (Videregående teoretisk, Videregående profesjon)
College/University (høyskolen/universitet)
Graduate/Professional (i.e. Master's/PhD)

42. Which of these categories best approximates how much you earn in a year, before tax (including student loans if you have any)? (Remember, your identity will be anonymous).
Less than 200,000 Kr
200,000-400,000 Kr
400,000-600,000 Kr
600,000-800,000 Kr
800,000-1,000,000 Kr
1,000,000 Kr or more

43. I am concerned about there being a lot of fat in my food.

I am not concerned at all

I am concerned a little

I am concerned

I am concerned a lot

I am extremely concerned

44. I am concerned about eating many calories.

I am not concerned at all

I am concerned a little

I am concerned

I am concerned a lot

I am extremely concerned

45. I am concerned about there being a lot of sugar in my food.

I am not concerned at all

I am concerned a little

I am concerned

I am concerned a lot

I am extremely concerned

46. I am concerned about gaining weight.

I am not concerned at all

I am concerned a little

I am concerned

I am concerned a lot

I am extremely concerned

Thank you for completing this questionnaire.
